# Virtual reality and augmented reality smartphone applications for upskilling care home workers in hand hygiene: a realist multi-site feasibility, usability, acceptability, and efficacy study

**DOI:** 10.1093/jamia/ocad200

**Published:** 2023-10-16

**Authors:** Norina Gasteiger, Sabine N van der Veer, Paul Wilson, Dawn Dowding

**Affiliations:** Division of Nursing, Midwifery and Social Work, The University of Manchester, Manchester, United Kingdom; Division of Informatics, Imaging and Data Sciences, Centre for Health Informatics, The University of Manchester, Manchester, United Kingdom; Division of Population Health, Health Services Research and Primary Care, The University of Manchester, Manchester, United Kingdom; Division of Informatics, Imaging and Data Sciences, Centre for Health Informatics, The University of Manchester, Manchester, United Kingdom; Manchester Academic Health Science Centre, The University of Manchester, Manchester, United Kingdom; Division of Population Health, Health Services Research and Primary Care, The University of Manchester, Manchester, United Kingdom; Division of Nursing, Midwifery and Social Work, The University of Manchester, Manchester, United Kingdom

**Keywords:** training, virtual reality, augmented reality, hand hygiene, long-term care, care home

## Abstract

**Objectives:**

To assess the feasibility and implementation, usability, acceptability and efficacy of virtual reality (VR), and augmented reality (AR) smartphone applications for upskilling care home workers in hand hygiene and to explore underlying learning mechanisms.

**Materials and Methods:**

Care homes in Northwest England were recruited. We took a mixed-methods and pre-test and post-test approach by analyzing uptake and completion rates of AR, immersive VR or non-immersive VR training, validated and bespoke questionnaires, observations, videos, and interviews. Quantitative data were analyzed descriptively. Qualitative data were analyzed using a combined inductive and deductive approach.

**Results:**

Forty-eight care staff completed AR training (*n* = 19), immersive VR training (*n* = 21), or non-immersive VR training (*n* = 8). The immersive VR and AR training had good usability with System Usability Scale scores of 84.40 and 77.89 (of 100), respectively. They had high acceptability, with 95% of staff supporting further use. The non-immersive VR training had borderline poor usability, scoring 67.19 and only 63% would support further use. There was minimal improved knowledge, with an average of 6% increase to the knowledge questionnaire. Average hand hygiene technique scores increased from 4.77 (of 11) to 7.23 after the training. Repeated practice, task realism, feedback and reminding, and interactivity were important learning mechanisms triggered by AR/VR. Feasibility and implementation considerations included managerial support, physical space, providing support, screen size, lagging Internet, and fitting the headset.

**Conclusions:**

AR and immersive VR apps are feasible, usable, and acceptable for delivering training. Future work should explore whether they are more effective than previous training and ensure equity in training opportunities.

## Background and significance

Hand hygiene is a simple infection prevention and control (IPC) behavior often practiced incorrectly by healthcare workers.^1^^,^^2^ Studies on nursing home workers have reported adherence to World Health Organization (WHO) hand hygiene guidelines from 6% to 27%.^3–5^ This is concerning given that appropriate hand hygiene is one of the most simple but effective means of preventing the transmission of harmful microbes and healthcare-associated infections, including antibiotic-resistant bacteria and COVID-19.^6^^,^^7^ Previous hand hygiene interventions have focused on acute care^8–10^ and have overlooked care workers.^3^^,^^11^ Various smartphone apps aim to improve hand hygiene, with some using virtual reality (VR) and augmented reality (AR).^12–15^ In VR, users wear a headset to feel immersed in a digital environment, while AR requires an overlap between the real world and the digital.

AR and VR apps for hand hygiene training afford learners opportunities that traditional learning approaches do not. For example, VR enables learners to interact with and problem-solve in context, given that it employs a three-dimensional simulated reality.^16^ AR hand hygiene apps can also facilitate psychomotor learning through repetition and testing, and encourage self-directed and purposeful practice.^15^ Lastly, when delivered on smartphone apps, the training can be easily accessible and use behavior change techniques like prompting practice (through push notifications) or personalization.^12^

Existing AR and VR hand hygiene technologies have been evaluated in hospitals^17^^,^^18^ and universities^15^ but not in care homes (which may include residential or nursing homes). The development of other VR training is also ongoing.^19^ Previous work has found that AR/VR training approaches are not always superior to conventional training^17^ and might not work for everyone equally, as those with more clinical experience may benefit less,^20^^,^^21^ and others may experience fatigue, cognitive load, stress, and cybersickness when using the technologies.^22–24^ This highlights a need to understand how, for whom, and in which contexts the technologies work. Furthermore, AR/VR hand hygiene interventions and implementing them in care homes may be complex as they will be delivered to learners with varying experience and in organizations with different policies and infrastructure. Lastly, implementation and uptake require careful consideration to determine which technology (AR or VR; immersive or non-immersive) is appropriate for each care home and the learners within them, which requires awareness of the available infrastructure and personal preferences.^25^

We are undertaking a program of work that seeks to understand how, for whom, and in which contexts or circumstances AR/VR can be used to upskill care home workers in hand hygiene practice. The program takes a theory-based perspective, whereby evidence reports on the processes that can lead to outcomes and prerequisites and contexts/circumstances for the intervention are considered. The process is iterative, consisting of developing, testing, and refining a program theory. We take a realist approach,^26^ which is theoretically driven, based on the principles of critical realism and considers that some interventions are complex and do not work for everyone equally. Realist evaluations therefore focus on understanding the mechanisms that are triggered by interventions and required to lead to outcomes.

Previous phases included a systematic review of 90 hand hygiene apps to identify high-quality existing apps that use AR/VR^12^ and a realist review of 80 papers to form a program theory on AR/VR training for healthcare workers which was tested and refined with 46 empirical studies.^27^ Lastly, interviews with 25 care home staff helped to refine the program theory further and understand implementation considerations.^25^ Through an iterative and systematic process various mechanisms have been identified as crucial for learning hand hygiene. These include repeated practice, task realism (ie, perceived relevance to work), feedback and reminding, and interactivity.

The current study forms the next phase in our program, where we implemented the training in a sample of care homes to address questions regarding intervention content and delivery, acceptability to learners and training completion, and fit within care homes. The purpose is to justify undertaking a full-scale evaluation^28^ and, while not the main focus, gather evidence on short-term/immediate outcomes to suggest potential effectiveness.^28^ Additionally, the work will fill a gap in research on hand hygiene training in care homes and on the feasibility, usability, acceptability, and real-world experiences of implementing AR/VR training in care homes. It will also help to explain how the technologies promote learning, by providing evidence for the mechanisms.

## Objective

This study aimed to assess the feasibility and implementation, usability, acceptability, and efficacy of VR and AR smartphone apps for upskilling care home workers in hand hygiene. A secondary objective was to explore mechanisms for learning triggered by the apps.

## Materials and methods

### Design

A realist mixed-methods study with pre- and post-test measures was conducted. The University of Manchester’s Proportionate University Research Ethics Committee approved the study (Reference: 2022-14903-24944). Participants provided written consent. The study aligns with the Good Reporting of a Mixed Methods Study checklist^29^ and the Realist And Meta-narrative Evidence Syntheses: Evolving Standards guidelines.^30^

### Setting, sample, and recruitment

We recruited a sample of care homes in Northwest England, United Kingdom with consideration of varying characteristics. The homes were identified from their websites and from our previous study^25^ in which we identified managers through networks of colleagues and the Care Quality Commission website. Managers recruited care workers (assistants or seniors) who spoke English and were older than 18 years. Participants were compensated with a £20 voucher.

We aimed to recruit up to 50 participants, with a minimum of 12 using AR and 12 using VR, which is considered sufficient for pilot and feasibility studies.^31^

### Procedure

**Figure 1.**
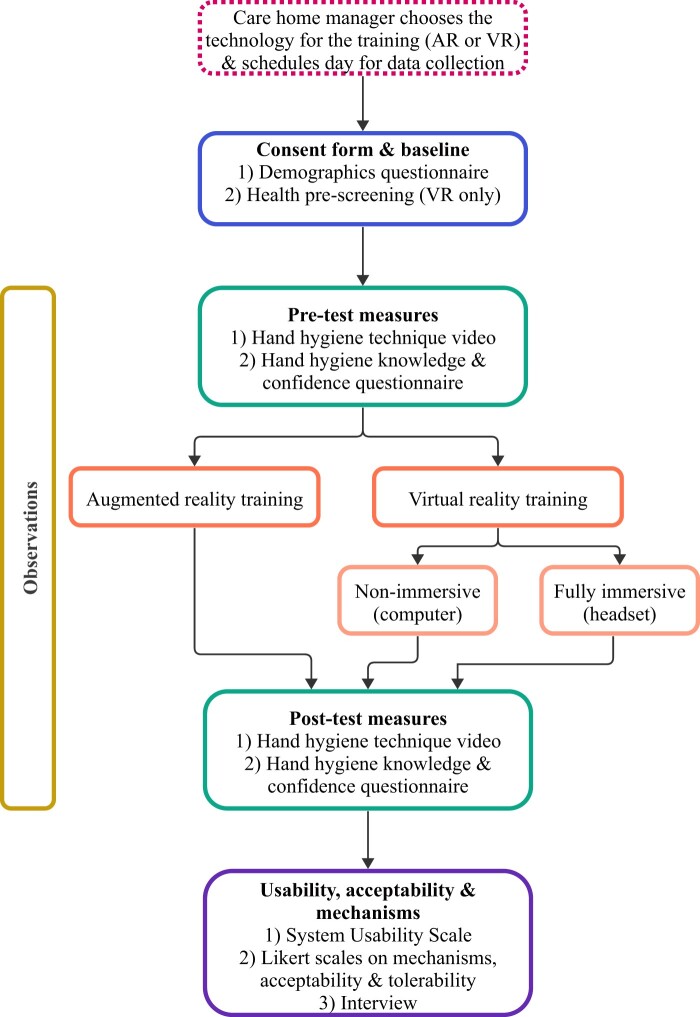
Overview of the data collection procedures.

Data collection occurred in-person at the care homes across 9 days between October and December 2022. The researchers helped care home managers choose which technology to deliver the training (AR or VR) by considering their preferences and access to the Internet and a computer. Participants first gave informed consent and completed the baseline and pre-test measures (Figure 1). This included providing demographic information (age, gender, ethnicity) and their role, experience working in care homes, previous hand hygiene training, and AR/VR use. They also answered 2 questions about hand hygiene compliance and 2 common reasons for poor practice, inspired by Baier et al.^32^ Participants then completed the training, followed by post-test and acceptability measures. Finally, we conducted semi-structured interviews and made observations throughout.

### Measures and analysis

Informed by our interview study,^25^ the primary outcomes were feasibility and implementation, usability, acceptability, and efficacy of the AR/VR training, while the secondary outcomes were the mechanisms of repeated practice, task realism (ie, perceived relevance to work), feedback and reminding, and interactivity. Table 1 shows how these were measured.

**Table 1.** Outcomes, mechanisms, and details of measures used.

Outcome domain	Specific outcomes	Measures	Details of measures
Feasibility and implementation	Recruitment and technology choice	Choice of technology	Number of care homes that participated. Choice of technology (AR or VR) by care home managers
	Uptake	Uptake	Proportion of participants who completed the training session they were offered. Reasons for non-uptake included personal preference or health reasons which prohibited engagement with the immersive VR training. This was measured through a health pre-screening form for VR headsets.
	Session and training completion	Session and training completion	Proportion of participants completing the training and session. Average time to complete the training.
	Training engagement; implementation considerations	Reactions to and views on the training, implementation, contextual considerations, and challenges (observations and interviews)	Interviews were transcribed verbatim. Participants could review their transcripts as a form of member-checking. Combined inductive/deductive approach. Interview and observation schedules are available in the Supplementary Material.
Usability	Usability	System Usability Scale	10-item System Usability Scale (SUS).^33^^,^^34^ Scores range from 0 to 100. Scores below 50 indicate poor usability, 70+ indicate good usability, and over 85 reflect excellent usability.^36^
		Reactions to and views on the training and challenges experienced (observations and interviews)	Combined inductive/deductive approach. Observation schedule is available in the Supplementary Material.
Acceptability	Learner satisfaction^a^; tolerability	Bespoke questionnaire (focus on learner satisfaction) and tolerability	1 Likert scale question with 5 responses (strongly disagree to strongly agree) asked whether they would support further work of this technology in their training. 2 questions assessed the tolerability of the training by asking whether the session was ended early due to side effects (yes; no) and the presence of 11 symptoms from the Simulator Sickness Questionnaire^38^ (Supplementary Material). The responses were counted, to understand how tolerable the training was and differences between the training.
		Reactions to the training (observations)	Combined inductive/deductive approach. See the Supplementary Material for the observation schedule.
Efficacy	Hand hygiene knowledge^a^; hand hygiene confidence	Hand hygiene knowledge questionnaire; Likert scale questions on confidence	Brief questionnaire (see Supplementary Material). 7 questions adapted from WHO questionnaire on hand hygiene for healthcare workers.^39^ Correct answers were summed. Higher scores indicated greater knowledge. 4 bespoke Likert scale questions with 5 responses (strongly disagree to strongly agree) explored confidence.
	Hand hygiene skill^a^	Videos of technique/skill	Participants demonstrated their technique using sanitizer. These were video-recorded and assessed for alignment with the WHO technique^37^ using an 11-item checklist.
	Skill	Overall scores (objective performance data)^b^	Overall compliance (0%-100%) and a scorecard of missed instances was recorded by taking screenshots of the VR app or computer screen. After each level in the AR app, the time to complete each level (lower times indicated better skill performance), pass/fail and which poses they had problems with were recorded.
Mechanisms	Perceived task realism (relevance to work)^c^; interactive learning^c^; feedback and reminding^c^	Likert scale questions on mechanisms; elaborated on in interviews	3 Likert scale questions with 5 responses (strongly disagree to strongly agree) about the relevance of the training task to their jobs, the interactivity of the learning experience, and the technologies ability to give feedback and remind learners of best practice (see Supplementary Material). Interviews covered the mechanism in more detail (see Supplementary Material).
	Repeated practice^c^	Repetitions; interviews	Objective performance across 3 repetitions (3 patients for the VR or 3 levels from the AR) taken from the systems. Interviews covered the mechanism in more detail (see Supplementary Material).

a Outcomes.

b Presented in the Supplementary Material as the data do not pertain to the whole participant sample.

c Mechanisms identified from our previous work.

We analyzed the quantitative data descriptively using IBM SPSS (v.27) (see Table 1 for further detail). The 10-item System Usability Scale (SUS)^33^^,^^34^ measured the usability of the technologies. Similar methods to a paper by Dowding et al.^35^ were used to analyze the SUS data. Responses were first converted to range from 0 to 4, with 4 indicating the most positive response. They were then summed and multiplied by 2.5, to give a score out of 100. Descriptive statistics were generated. The scale scores ranged from 0 to 100, with scores below 50 indicating poor usability, over 70 indicates good usability, and scores over 85 reflect excellent usability.^36^

The videos of the hand hygiene technique (before and after the training) were assessed for alignment with WHO best practice guidelines^37^ using an 11-item checklist based on the 6 poses, the length of the practice (minimum of 30-40 s) and whether the hand surfaces were covered with enough sanitizer. Each item was scored 1 if present and 0 if absent. The items were summed, with higher scores indicating better technique. One researcher (N.G.) first rated 20 videos (21%). A second rater who was blinded to the technology/intervention groups rated the same videos. Inter-rater reliability was calculated using a Cohen’s Kappa on SPSS and indicated substantial agreement between the raters: *κ *= 0.79 (95% CI, 0.711-0.871), *P *<* *.001. Consensus was reached through discussion, and this informed the rating procedure for the remaining videos.

Similar to our previous study, we used NVivo (version 12.7.0) and a combined deductive and inductive approach to analyze the qualitative data, consisting of the interviews and observations.^25^ The mechanisms identified in our previous work were used for the deductive approach. New themes were identified from the data pertaining to implementation considerations and reactions to the technology. The researcher who collected the data (N.G.) analyzed all the data by adding, changing, and moving codes while a second (D.D.) double-coded the data for 10 participants (21%). Disagreements regarding interpretation and the codes were discussed until consensus was met. The final themes were then determined and represented by interview quotes or observational notes. Inter-coder reliability was calculated on NVivo using a Cohen’s Kappa and showed substantial agreement κ = 0.72 (raw agreement rate: 96.04%).

### Intervention (training)

Informed by our previous interview study,^25^ the intervention/training was a short “refresher session” of hand hygiene best practice. A similar approach was taken when using VR to train hospital staff.^17^ The intervention functions included:

- Education: increasing knowledge/understanding of when infections spread and of best practice guidelines. This may influence psychological capability and reflective motivation.
- Training: imparting skills that align with best practice hand hygiene which may impact a learner’s physical and psychological capability, physical opportunity, and automatic motivation (as practice becomes subconscious).
- Modeling: providing examples for imitation to impact automatic/subconscious or reflective/conscious motivation.

As the training needed to be suited to the context, 2 technology options were used: (1) Tork VR Clean Hands Training app in a headset,^13^ with a non-immersive option and (2) SureWash.^14^ The apps were selected from our review of 90 hand hygiene apps as they were among the highest scoring.^12^ The training took approximately 15 minutes to complete.

A researcher with behavior change expertise gave feedback on the intervention, measurement tools, and study design. A member of the public tested the AR intervention and outcome measures, giving feedback on understanding and readability. A care home worker, a registered nurse with formal IPC training and an individual who provided home care provided feedback on the interventions, specifically the care and IPC aspects.

#### VR training

**Figure 2.**
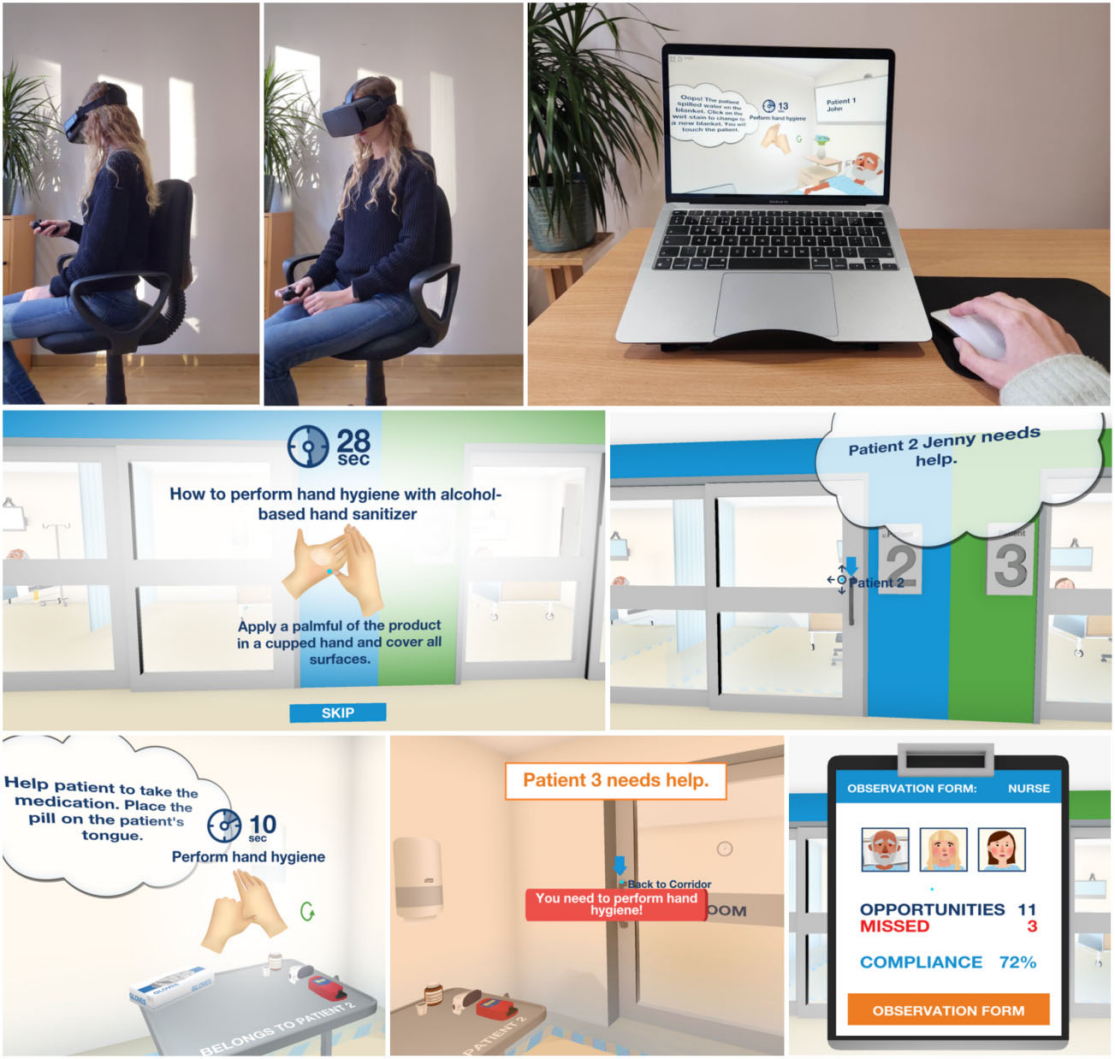
Images showing the seated position for the immersive VR training (top left and middle), non-immersive VR set-up (top right), and screenshots from the game^13^ (middle and bottom row).

The VR groups were screened for health problems or preference to determine whether the headset was suitable. If unsuitable, non-immersive training was completed on a laptop on a stand (for ergonomic optimization). Learners navigated the game via the Bluetooth mouse (Figure 2). For the immersive training, a smartphone with the Tork VR Clean Hands Training^13^ app was placed in the DESTEK V5 headset. Participants were seated to avoid bumping into objects or tripping (Figure 2). They wore a disposable VR mask under the headset and used a Bluetooth remote and “swiveled” to navigate by moving the head and upper body.

Both options delivered the same training. The app demonstrated the WHO’s 6 steps for hand hygiene.^37^ Learners then played a nurse and cared for 3 patients in the game, identifying moments for hand hygiene from the WHO 5 Moments for Hand Hygiene.^37^ A patient alarm prompted learners to move to the next patient. Feedback included reminders for missed hand hygiene moments, when to use gloves and how to dispose of them and medical waste. A compliance score and summary of the missed instances were presented upon completion.

#### AR training

**Figure 3.**
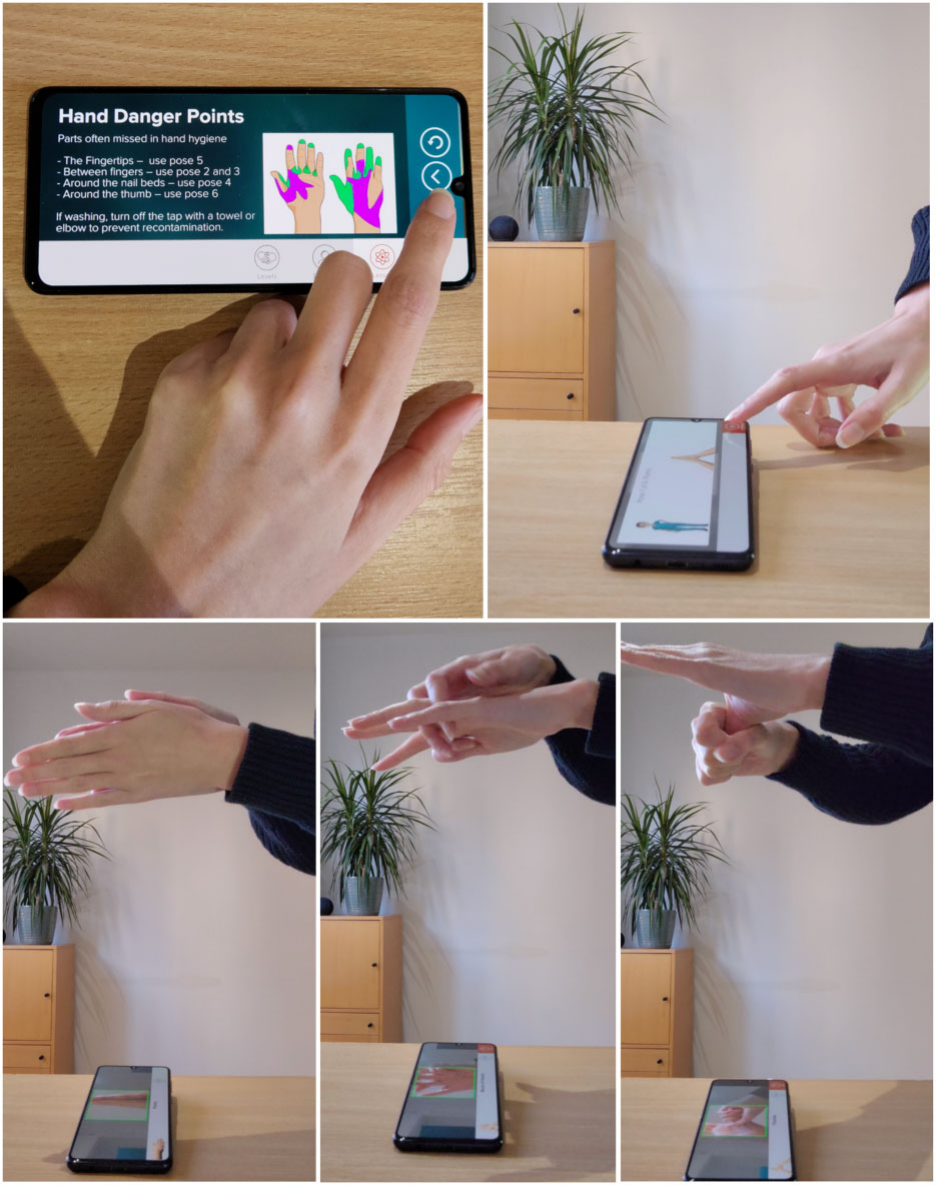
Image of the SureWash (GLANTA)^14^ AR app lesson (top left), poses lesson (top right), and training (bottom row).

We used the premium SureWash^14^ (GLANTA) app. Participants read the educational content (lesson), which explained the importance of hand hygiene, WHO 5 Moments for Hand Hygiene and the correct technique. The smartphone was placed on a table, and learners practiced the technique above the device, which used a motion-sensing algorithm to detect movement (Figure 3). Level 1 guided learners through the WHO 6 steps^37^ and gave feedback on switching hands. Level 2 taught how to build a smoother flow by putting the poses together. This was completed twice to record changes over repeated attempts. Level 3 asked learners to perform the poses in under 40 seconds. After each level, the app gave a pass/fail and reported challenging poses and the overall time to complete the poses.

## Results

### Feasibility and implementation

#### Care home recruitment and technology choice

Five care homes participated in the study. All were privately owned, with 3 belonging to a chain organization (see Table 2). Three were in neighborhoods with the highest levels of deprivation (Indices of Multiple Deprivation^40^ rating of 1), and 2 were in less deprived neighborhoods (7 and 9, respectively, of 10). One home experienced Internet issues during the study, but all owned computers and iPads. In one, staffs were allowed to use smartphones during quieter days for training. Another only allowed seniors to use smartphones to communicate with management.

**Table 2.** Summary of the care homes and participants.

Care homes (*N* = 5)	Number (%)
**Type** ^a^	
Nursing	2 (40)
Residential	1 (20)
** **Both	2 (40)
**Size**	
Medium (average of 31 beds, range 20-49)	4 (80)
Large (60 beds)	1 (20)
**Care Quality Commission rating**	
Not inspected	1 (20)
** **Good	3 (60)
** **Requires improvement	1 (20)
**Previous VR use**	
** **Yes (eg, falls or dementia training, therapy for residents)	3 (60)
** **No	2 (40)
**Participant characteristics (*N* = 48)**	**Mean (SD), range**
**Age**	38.10 (±13.57), 18-67
	**Number (%)**
**Gender**	
Female	41 (85)
Male	7 (15)
**Ethnicity**	
** **White/British	33 (69)
Black, African, Black British, or Caribbean	9 (19)
Any other ethnic group	3 (6)
Mixed or multiple ethnicities	2 (4)
** **Asian or Asian British	1 (2)
**Role**	
Care assistant^b^	32 (67)
Senior/advanced carer^c^	13 (27)
Both (care assistant and senior/advanced carer as needed)	3 (6)
**Work experience**	
Experienced (more than 12 months experience)	37 (77)
Novice (less than 12 months experience)	11 (23)
**Previous VR use**	
** **None	21 (44)
** **Have used VR	27 (56)
Entertainment purposes (eg, 5D horror movie or games)	16 (59)
Training at the care home (eg, dementia awareness or falls)	11 (41)
Other (eg, care show, gym/exercise, or career showcase)	3 (11)
**Previous AR use**	
** **None	32 (67)
** **Unsure	3 (6)
** **Have used AR	13 (27)
Entertainment purposes (eg, Pokémon Go game)	13 (100)
**Received hand hygiene training**	
** **No	4 (8)
** **Unsure	1 (2)
** **Yes	43 (90)
** **Formal training through care home	33 (77)
** **Formal training through an external company	11 (26)
** **Informal training through managers or senior staff	14 (33)
**Tools used for hand hygiene training (if received)**	
** **Videos	25 (58)
** **Glitterbug (UV light with gel)	20 (47)
** **Photos	17 (40)
** **Text descriptions	13 (30)
Posters	12 (28)
Video-conference session (eg, Zoom, Skype, or Teams)	5 (12)
Other (eg, online modules, TV advert, and smartphone app)	7 (16)
**Hand hygiene has been monitored since starting work at care home**	
** **Yes, informal monitoring (eg, spot-checks)	30 (63)
** **Yes, formal monitoring (eg, Care Quality Commission, or checklists)	11 (23)
No	7 (15)
Unsure	3 (6)
**Most common reasons for hand hygiene compliance in the care home**	
Forgetting to practice hand hygiene	34 (71)
Lack of knowledge of best practice (when/how to perform hand hygiene)	33 (69)
Hand hygiene causes skin irritation	14 (29)
Not having time to practice hand hygiene	13 (27)
Too much effort to practice hand hygiene	2 (4)

a Nursing and residential homes provide 24/7 care and support. Nursing homes provide higher levels of medical care as qualified nurses are available on-site.

b Care assistants are unregistered staff who provide care-related tasks such as dressing, washing, and physical support. They may also perform domestic duties like food preparation and emptying bins.

c Senior care staff delegate tasks and have other responsibilities (eg, administering medications and record-keeping). Abbreviation: SD, standard deviation.

Two care homes chose VR to try a different approach to training, while 3 chose the AR training for convenience and infrastructure reasons, as it does not require a computer or Internet.

#### Uptake, session and training completion, and engagement

**Figure 4.**
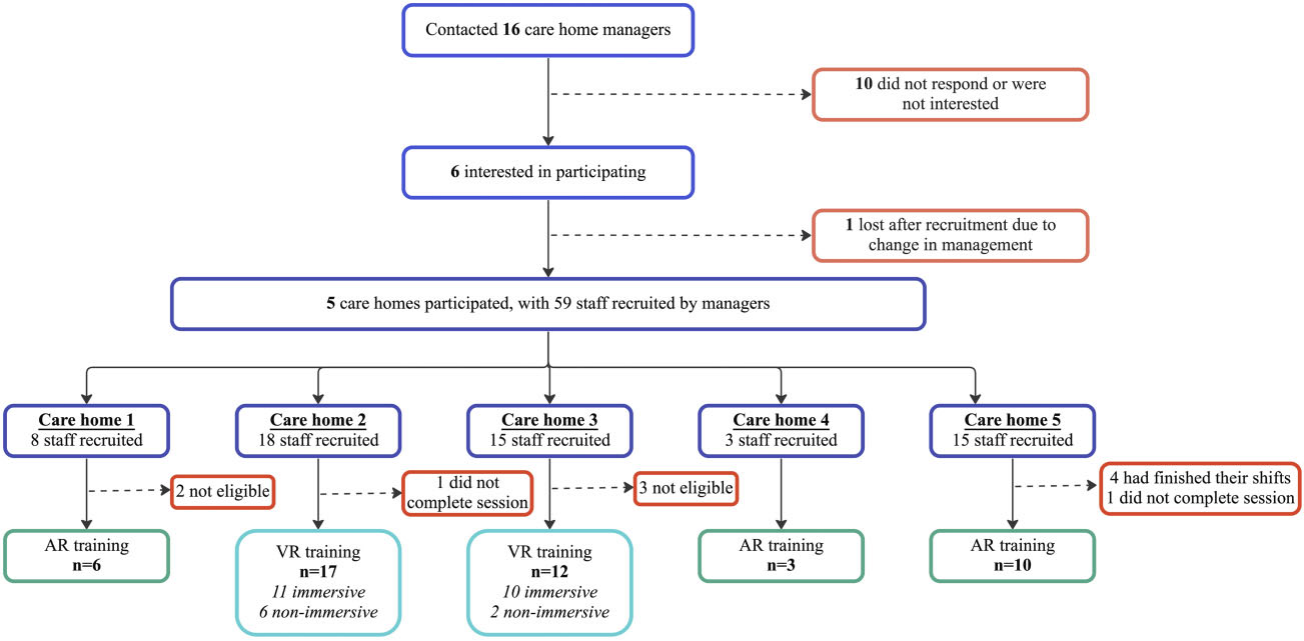
Flowchart presenting care home and participant recruitment process.

Fifty staff members were enrolled in the training, but 2 did not complete the session due to time constraints so were ultimately excluded from the analysis (see Figure 4).

Of the 48 participants, 41 were interviewed. On average, the interviews lasted 4.23 min (±1.16; range 2.08-8.20). There were approximately 36.5 hours of observations.

As summarized in Table 2, the 48 participants had an average age of 38.10 years (±13.57), ranging from 18 to 67 years, with 15% male and 25% identifying as Black, Asian, or mixed ethnicities. This is similar to England’s adult social care workforce, where the mean age is 45 years, 18% are non-female, and 23% have Black, Asian, or minority ethnicity.^41^ On average, participants had 5 years and 8 weeks experience working in care homes (range: 3 weeks to 22 years). The majority (56%) had previous experience of VR in their personal or professional lives. Ninety percent had received hand hygiene training and 63% had their hand hygiene monitored informally. On average, participants estimated hand hygiene compliance at their care homes at 89% (±12.6, range: 60-100%). The 2 most common reasons for non-compliance were forgetting and lack of knowledge of best practice.

Uptake was defined as the proportion of participants who completed the training session they were offered. There was 100% uptake for the AR training, as all 19 staff who were offered the AR training completed it. Twenty-nine participants were offered the immersive VR training, however only 21 participants completed the immersive version, as the remaining 8 were unable to use the VR headset due to health reasons. Uptake for the immersive VR training was therefore 72%. All 8 participants completed the non-immersive training after being offered it (100% uptake).

Sessions took 45.58 min on an average (±10.39, range 28-79), of which the training took 12.10 min (±3.37 range 7-21). The VR training took longer (VR mean: 13.07, ±3.7, range: 8-21; AR mean: 10.63, ±2.09, range: 7-16).

#### Training engagement and implementation considerations

For the AR training, 47% (*n* = 9) of participants were observed skim-reading the lesson content, while 26% (*n* = 5) used the “help” button to get assistance, and 21% (*n* = 4) stood up for the training. For the immersive VR training, 29% (*n* = 6) read the instructions aloud, spoke to the patient characters, or narrated their gameplay. Four (14%) held the headset to stabilize it. A stress/panic response was observed during the patient alarms, whereby 48% (*n* = 10) for the immersive VR training and one in the non-immersive training moved quicker or verbalized surprise. One yelled, “*I’m coming patient 3.*”

Support by managers and senior carers was crucial for recruitment and scheduling. For the immersive VR training, the space was vital for safety reasons, as some participants moved forward and needed to be moved away from furniture. Interruptions were frequent, and many sessions were paused due to staff, residents, and other activities (eg, COVID-19 vaccines for staff).

From the interviews (Table 3), participants identified characteristics that may limit the uptake of the training, including limited English, health issues (eg, nausea), claustrophobia or poor eyesight, and limited digital skills. Participants suggested encouraging potential non-users, providing step-by-step instructions, health pre-screening, and a training exercise for the VR headset.

**Table 3.** Supporting quotes for individual-level implementation considerations for AR and VR hand hygiene training.

Considerations	Supporting quotes from interviews
Learners with health issues or limited digital literacy	Certain people won’t be able to use that training due to either illness or different reasons like nausea or sickness and things like that. So obviously that’s got to be a challenge trying to roll that out to the people. And obviously people that are not very good with technology may struggle a bit. (Participant 11, immersive VR training)
Language barriers	There will be a little bit of language barriers as well if you, you know, get in contact with staff who probably are not like 100%, you know, there is some language barriers, then you may struggle a little bit. So, it will probably take longer than you would expect, you know, to complete the task. (Participant 30, AR training)
Supporting implementation through a training exercise	Give them practice with it… instead of bringing them in for the initial training, just say okay, here’s like five, ten minutes to have a play around, get the feel for it and then we’ll begin. (Participant 26, immersive VR training)

Technology-related issues were observed whereby some participants commented on the small screen size and attempted to make the AR content bigger by zooming. One suggested using an iPad instead. Some participants struggled to fit the headset due to their hairstyles or smaller head sizes. When the Internet disconnected, the non-immersive VR training lagged and lost audio.

### Usability

The immersive VR and AR training had the highest mean SUS scores at 84.40 (±8.66, median: 85) and 77.89 (±15.62, median 77.5), respectively, indicating good usability. The non-immersive training had borderline poor usability, scoring 67.19 (±22.46, median: 71.25).

For the AR training, 9 participants (47%) needed prompting to keep their hands in the frame and use the app (eg, changing poses). Four (21%) were observed struggling with the fingertips pose, which the app struggled to detect: *I was doing the correct motion, but the app was struggling to pick it up…But overall, I feel like the app works well. (Participant 36, AR training).*

For the immersive VR training, 11 participants (52%) needed prompting to navigate the training and unfamiliar tasks (eg, wound care). For the non-immersive training, 7 participants (88%) had issues navigating the game and commented on the sensitivity and difficulty moving around in the digital hospital. Most overcame these problems as they progressed through the game: *I think when you first start off it is really hard to get the gist of how it’s working, like, when I thought the arrow, I didn’t know it was the blue dot we were following, I thought it was the arrow really…by the third patient I did get the hang of it but it took me three patients to get there. (Participant 14, non-immersive training).*

### Acceptability: learner satisfaction and tolerability

Participants were satisfied with the training, with 90% (*n* = 43) agreeing or strongly agreeing that they would support further work of the technology in their training. Scores were highest for the immersive VR (mean: 4.67 of 5, ±0.58) and AR training (mean: 4.37, ±0.60), whereby 95% agreed or strongly agreed with the statement. Scores were lowest for the non-immersive VR training; only 63% agreed or strongly agreed with the statement (mean: 3.75, ±1.04).

No-one stopped due to discomfort or side effects. Only 5 (10%) reported feeling side effects, of which 3 completed the AR training. Side effects included fatigue (AR *n* = 2), tired shoulders/arms (AR *n* = 2), difficulty concentrating (AR *n* = 1), eye strain (non-immersive VR *n* = 1), dizziness (immersive VR *n* = 1), and stomach awareness (immersive VR *n* = 1).

We observed 3 participants in the AR group displaying discomfort from holding their hands above the smartphone, evidenced by shaking their arms and commenting on minor discomfort. For 3 others, dry skin was observed on the device and table from rigorous rubbing. One participant reported mild dizziness during the immersive VR training from moving between rooms in the game.

### Efficacy

#### Hand hygiene knowledge

Participants initially scored an average of 65% on the knowledge questionnaire (mean: 11.71, ±2.14; maximum 18), increasing to only 71% after the training (mean: 12.79, ±2.44). The immersive VR group scored an average of 73% (mean: 13.05, ±2.36), and the AR and non-immersive VR groups scored 70% and 69%, respectively (AR mean: 12.63, ±2.67; non-immersive VR mean: 12.5, ±2.33).

After the training, no-one could recall all 5 Moments for Hand Hygiene. Seventeen participants mentioned hand hygiene around other tasks, such as before food preparation, feeding and laundry, and after emptying bins. Some mentioned after sneezing or coughing (*n* = 5) and using the toilet (*n* = 7). Only 52% (*n* = 25) correctly stated that hand hygiene should be performed before and after wearing gloves. However, 73% (*n* = 35) identified the ideal length of hand hygiene compared to 56% (*n* = 27) before the training. Majority (85%, *n* = 41) also identified healthcare workers’ hands as the main route of cross-transmission of potentially harmful germs between patients.

#### Hand hygiene confidence

**Figure 5.**
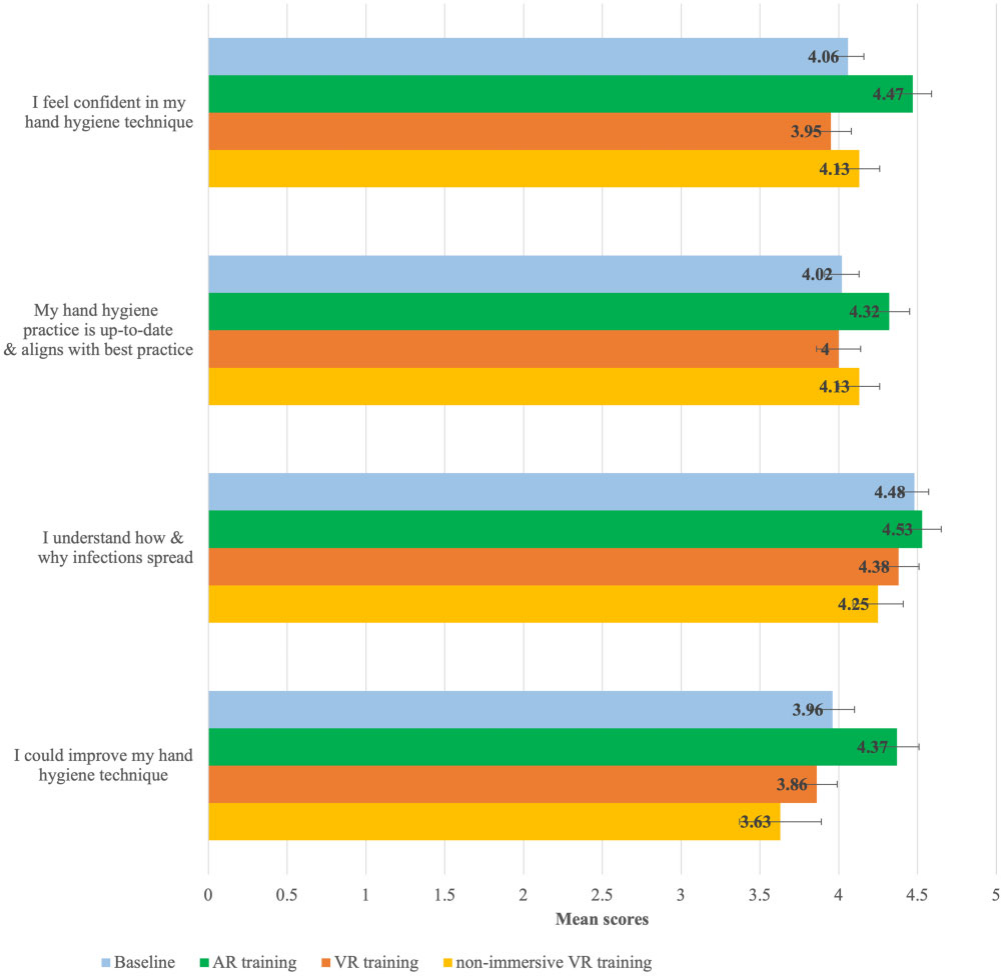
Clustered bar graph showing mean confidence scores at baseline and after the training.

Figure 5 shows the average scores per group for the confidence questions, compared to baseline. Almost everyone (96%, *n* = 46) agreed or strongly agreed that they felt confident in their hand hygiene technique after the training, compared to 85% (*n* = 41) before. The AR and non-immersive VR groups reported the highest scores after the training. Unlike the AR group which reported the most improvement in confidence after the training, the immersive VR group reported less confidence. In fact, their confidence decreased from baseline.

The majority (79%, *n* = 38) agreed or strongly agreed that their hand hygiene practice is up-to-date and aligns with best practice, increasing to 94% (*n* = 45) after the training. Mean scores after the AR and non-immersive VR training improved, while the scores after the immersive VR training decreased slightly.

There was no change to the number of participants who agreed or strongly agreed that they understood how and why infections spread (98%, *n* = 47 before/after training). Only the AR group scored slightly higher after the training.

Lastly, 81% (*n* = 39) agreed or strongly agreed that they could improve their hand hygiene technique compared to 85% (*n* = 41) after the training. Perceived improvement was highest in the AR group.

#### Hand hygiene skill

Overall, average skill scores increased from 4.77 (of 11) (±1.80) to 7.23 (±2.31) after the training. We noticed improvement in 79% (*n* = 38) of participants. These were highest in the AR group (AR: 4.58-8.32; immersive VR; 4.90-6.71; non-immersive VR: 4.89-6.71). Of the 10 participants who did not improve, 4 did not change their scores, and 6 had lower scores after the training. Six of the non-improvers were in the immersive VR group, 3 were in the non-immersive VR group, and 1 was in the AR group.

**Figure 6.**
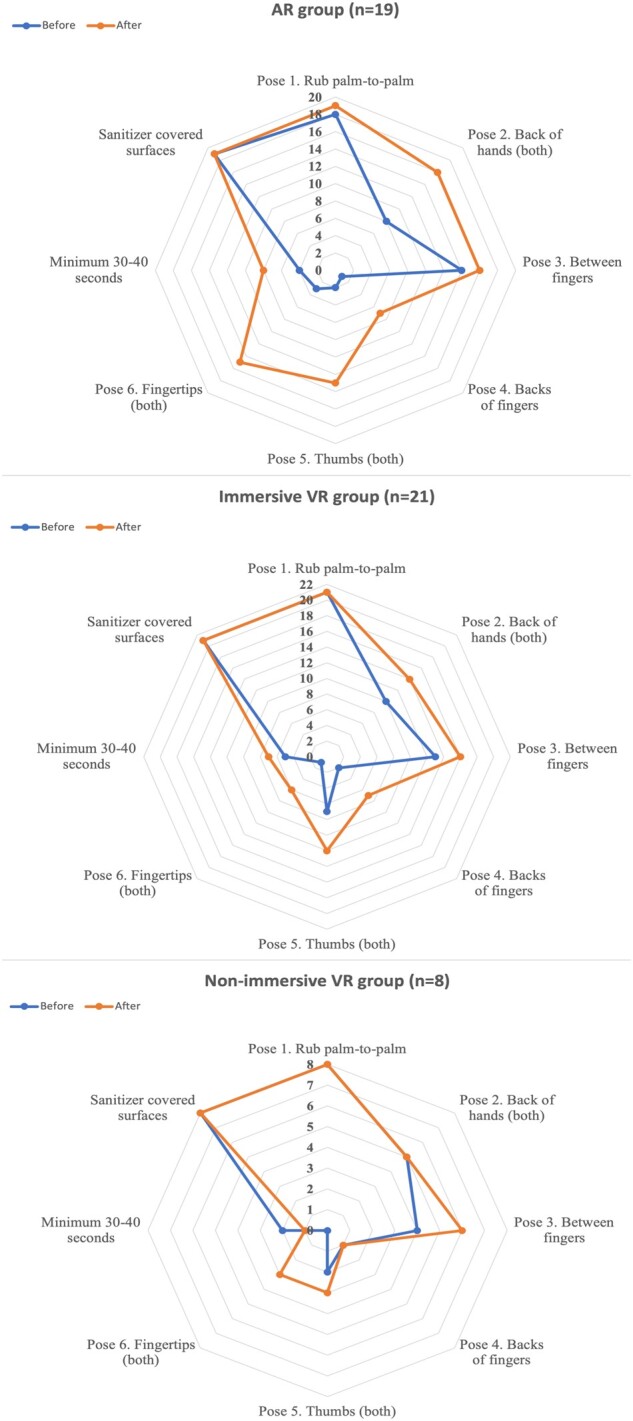
Radar charts showing how many of the learners met the hand hygiene technique criterion before and after the training, per intervention group.

Figure 6 shows the number of learners achieving the skills for each intervention group. Everyone used enough sanitizer to cover their hands before and after the training. Major improvements were seen in the fingertips and the thumbs poses, whereby an additional 20 and 17 care staff, respectively, performed these poses after the training (fingertips: 4 before, 24 after; thumbs: 11 before, 28 after). The specific length of the technique improved in 77% (*n* = 37), increasing from 22 (±9.40) to 26 seconds (±10.41) on an average.

### Mechanisms

Perceived task realism (relevance to work), interactive learning, feedback and reminding, and repeated practice were all considered important mechanisms for learning by participants, which the training triggered (see Table 4).

**Table 4.** Supporting data and interview quotes for the mechanisms triggered by the hand hygiene training.

Mechanisms	Quantitative results	Qualitative results	Supporting quotes from interviews
Perceived task realism (relevance to work)	96% (*n* = 46) agreed or strongly agreed that the task in the learning tool was relevant to their work (mean: 4.48, ±0.74). Scores were similar across the AR and immersive VR groups, with 95% (*n* = 18; *n* = 20) agreeing or strongly agreeing with the statement (AR mean: 4.53, ±0.61; immersive VR mean: 4.52, ±0.93). The average score was slightly lower for the non-immersive VR training (mean: 4.25, ±0.46).	**AR** Participants agreed that the AR training content was relevant. **Immersive and non-immersive VR** Everyone agreed that the hand hygiene content was relevant, but most were unfamiliar with the hemoglobin device and nursing tasks (eg, wound care). Participants identified differences to their work like that they always wear gloves when touching a resident, wear spit-guards, and that residents are mobile. Some suggested scenarios like food preparation, care, and managing bed sores better reflect their work.	The whole thing is relevant because you’ve got to wash your hands each and every time you go to a different person or whatever, whatever you’re doing. (Participant 3, AR training). Before we would touch any resident at all we would always put gloves on because you’d never know what you’re going to touch. So even if that resident is about to fall out of bed, it could be because they’ve slipped on some faeces for example. (Participant 21, immersive VR training)
Interactive learning	94% (*n* = 45) agreed or strongly agreed that the learning tool was more interactive than previous training (mean: 4.42, ±0.61). The immersive VR training was perceived most interactive (mean: 4.57, ±0.51), with 100% (*n* = 21) agreement. Most of the AR group (95%, *n* = 18) agreed or strongly agreed that it was more interactive (mean: 4.42, ±0.61). Only 75% (*n* = 6) of the non-immersive VR group agreed or strongly agreed with the statement (mean: 4, ±0.76).	**AR** Participants explained that they physically practiced the poses rather than looking at images or videos. They enjoyed the game component, feedback and said it felt more human and thorough, as it was not rushed and was conducted one-to-one in-person, unlike training on Zoom. One participant explained that while it was fun, learning at a sink or using the UV machine was more interactive. **Immersive VR** Participants described it as interesting, novel, modern, and fun. They felt like they were actually caring for patients. Everyone preferred it over reading, listening, and watching. **Non-immersive VR** Some likened it to Zoom, online modules, or videos. Others said it was more interactive and fun, which made it better than listening to others describe hand hygiene.	For me I think it was practising the motions, being able to do it practically. So, I know the World Health Organisation has the picture guide, but this makes it more interactive, and I feel like it’s good for learning purposes. (Participant 36, AR training) It was a lot more interactive than a video because you’re actually doing the task and it’s approving whether you’re doing it correct or not. (Participant 28, AR training) It was like the real world to me, you know, doing that instead of like somebody stood up at the front of a class or whatever talking to us. (Participant 8, immersive VR training). It’s either online training or paper training or like Zoom. It’s all very similar and it’s all very, it’s repetitive. It’s the same. (Participant 14, non-immersive VR training). Most of the time when you do like hand hygiene training, you watch a video of someone else washing their hands. And then you do a questionnaire and that’s it. It [non-immersive VR] gets you more involved and I think you’re more likely to remember something that’s, more interactive like what that was. (Participant 18, non-immersive VR training).
Feedback and reminding	Everyone (*n* = 48) agreed or strongly agreed that the learning tool gave them feedback and reminded them about good practice (mean: 4.52, ±0.51). Mean scores were similar in the AR (mean: 4.58, ±0.51) and immersive VR groups (mean: 4.57, ±0.51) but lower in the non-immersive VR group (mean: 4.25, ±0.46).	The tools gave participants feedback on what they were doing correctly and where to improve through prompting and scores/marks. The AR app also gave feedback on the length and challenging poses. Participants valued the final report card in the VR training and that they could not continue until they had followed the correct procedure. The feedback and reminders also facilitated reflection, for example the AR participants commented on the poses they normally forget (eg, fingernails). During the VR patient alarm, learners reflected that emergencies make it easy to forget hand hygiene. In the interviews, participants explained their realizations about their usual technique, such as that they forget poses or already do it correctly.	I’ve been taught it before, and I’ve done it plenty of times before but it’s easy to forget. But the actual technique and the step-by-step routine, that’s been refreshed. (Participant 28, AR training). I was able to see what I missed, what I didn’t miss, where I could improve. (Participant 20, non-immersive VR training) It wouldn’t let me press and that was because I’d not put gloves on. So it was just, it was a reminder. You couldn’t go any forward until you do this first which I found really helpful. (Participant 16, immersive VR training) Reflection: I think it’s consolidated that I do know when to change my gloves and I do know when I’m supposed to use the hand sanitiser. So, I’m right in what I preach to the rest of the staff. (Participant 21, immersive VR training) I definitely feel like I know how to wash my hands properly now. And I have been forgetting like some important parts of it such as fingernails. So, I do feel like I will be able to wash them thoroughly next time and after that going forward. (Participant 27, AR training)
Repeated practice	**AR** The app recorded how long it took to perform the 6 poses. First, the average time was 143 s (±35), with 95% (*n* = 18) improving at the second attempt (mean: 92 s, ±22). Only 47% (*n* = 9) improved at the third attempt (mean time: 95 s, ±26). Everyone passed the levels. **Immersive and non-immersive VR** Compliance scores were recorded for each patient cared for in the game. The 29 participants reached an average compliance score of 59% (±22.57) for patient one. 55% (*n* = 16) improved at the second patient (mean: 61%, ±16.46), and 86% (*n* = 25) improved at the third (mean: 90%, ±24.56) with 83% (*n* = 24) scoring 100%.	Everyone stated that repeated practice is essential to correcting hand hygiene technique, helping those who have forgotten and ensuring that skills are up-to-date. Accordingly, through repetition, skills can become “*routine*” or subconscious “*muscle memory*.”	Practice makes perfect as we say and in some instances with things like hand hygiene it is important to get it right. So yes, I would say the more I practice doing it the correct way, the more naturally it would come to me to do it the correct way rather than doing it what I think is correct or not thorough enough. (Participant 1, AR training) I suppose it’s like muscle memory. The more you do things, you just get used to it in the end, so you just do it more often. (Participant 11, immersive VR training)

Ninety-six percent agreed or strongly agreed that the task in the learning tool was relevant to their work. However, some of the content (not related to hand hygiene) in the immersive VR and non-immersive VR training was less relevant (eg, hemoglobin device and nursing tasks).

Ninety-four percent agreed or strongly agreed that the learning tool was more interactive than previous training with the immersive VR training perceived most interactive and the non-immersive VR group least interactive. Participants enjoyed practicing the poses in the AR training and the immersive VR training was described as interesting, novel, and fun. The non-immersive VR training was sometimes likened to Zoom or other online modules.

One hundred percent of participants agreed or strongly agreed that the learning tool gave them feedback and reminded them about good practice. However, scores were lower in the non-immersive VR group. This mechanism also facilitated reflection on poses that are easily forgotten or on usual technique.

In the interviews, everyone stated that repeated practice is essential to correcting hand hygiene technique, helping those who have forgotten and ensuring that skills are up-to-date. Skills can then become “*routine*” or subconscious “*muscle memory*.” The AR app recorded how long it took to perform the 6 poses. Ninety-five percent improved at the second attempt but only 47% improved at the third attempt. For the immersive VR and non-immersive version, compliance scores were recorded for each patient. Fifty-five percent improved at the second patient and 86% improved at the third with 83% scoring 100%.

## Discussion

This study has explored VR/AR smartphone apps for upskilling care home workers in hand hygiene. The immersive VR and AR training had good usability and acceptability, being perceived as more interactive than previous training and resulting in high learner satisfaction with support from 95% of learners. However, the non-immersive VR training was less acceptable, less interactive, and had borderline poor usability. Regarding efficacy, after the training, there were minimal improvements to knowledge. However, hand hygiene technique improved (especially for the AR group). Importantly, AR/VR triggered mechanisms including repeated practice, task realism, feedback and reminding, and interactivity, which are essential for learning. Lastly, we noted implementation considerations, including managerial support, the space available, support for potential non-users, screen sizes, lagging due to poor Internet connections, and fitting the headset.

Leadership and collaboration are crucial for facilitating the implementation of AR/VR training,^42–45^ with care home managers and senior carers playing an important role in training engagement and choosing which technology is best suited to their setting.^25^ However, it is vital that the considerations regarding using the VR headset are clearly outlined to managers as it requires more infrastructure, funding, and technical support. It is especially important that the headset is fitted properly and Internet issues are resolved for learner comfort, as a poorly fitting headset, flickering screen, or lagging/latency may induce cybersickness.^46^ These additional requirements may encourage managers to use the AR training instead.

Ideally, short refresher sessions for hand hygiene training should be introduced, which can be repeated for new and temporary staff. Self-directed training may be appropriate given that staff may already own devices that can be used; with smartphone penetration estimated to reach 95% of the UK population by 2025.^47^ Short sessions that can be repeated also account for the transient nature of the care staff workforce as staff may work in multiple care homes or on shifts where training and monitoring are unavailable. In 2021 and 2022, 24% of England’s adult social care workforce was employed on zero-hours contracts, and employers struggled to recruit and retain their staff, resulting in competition and movement.^41^

Our results regarding improved technique and learner satisfaction were consistent with research on AR/VR hand hygiene training for other learners. In one study, the SureWash AR system was implemented in an Irish hospital with poster reminders, an adenosine triphosphate monitoring system, auditing through observation and verbal feedback.^18^ SureWash demonstrated the correct technique and assisted with auditing. Overall, this approach significantly improved technique and compliance. SureWash alone also led to compliance in 81% of 47 university students after an average of 24 training sessions.^15^ Another study compared VR training to lectures in German hospitals, finding that 69% of the 81 healthcare workers preferred VR.^17^ However, it did not result in higher compliance with disinfectant use. While AR/VR may deliver effective hand hygiene training, it remains unclear whether this translates to longer term impacts and how many sessions are required for optimal learning.

The non-immersive VR training was less usable, acceptable, and had lower efficacy compared to the other training modalities. It is possible that digital literacy skills influenced the usability of the technologies, while the novelty and motivation of the AR and immersive VR technologies may have influenced the outcomes. For example, a previous RCT with 66 participants learning neuroanatomy found that while using a novel immersive VR system was just as effective as educational textbooks, it was significantly more engaging, enjoyable, useful, and motivating for learners.^48^ The ARCS Model of Motivational Design further explains the importance of motivation for learners, stating that this consists of attention, relevance, confidence, and satisfaction.^49^ Specifically, learners must be interested in the topic, understand the relevance to their personal goals/motives, and feel confident in their ability to learn. The construct of “relevance” aligns with our mechanism of “perceived task realism (relevance to work)” which is important for hand hygiene training in care homes.^25^ It may be possible that the novel technologies were more engaging and interactive, thus facilitating the other constructs of motivation and resulting in better outcomes, compared to the computer-based version.

The lower usability and efficacy of the non-immersive VR training may result in inequitable training opportunities if only the immersive VR training was rolled out, as 28% of the VR participants could not use the headset for health reasons. However, offering the AR training as a non-immersive alternative (rather than the non-immersive VR training) would ensure that staff who cannot use VR headsets could still engage in effective and acceptable training. Differences in the training also helped explain reflection as an essential part of the feedback and reminding mechanism and potentially the reason for adverse outcomes. Participants in the immersive VR group had less confidence in their technique, and those in the AR group were more likely to perceive a need for improving their technique. As suggested in the qualitative data, learners may have realized what they were doing wrong by reflecting on their usual practice during the training (education and modeling), resulting in less confidence.

Our findings clearly indicated that future development of VR/AR training should focus on applying the WHO 5 Moments for Hand Hygiene to care homes and re-educating staff on wearing gloves. The 5 Moments only partially translate to care homes due to environmental differences. Teesing et al.^50^ explained that hand hygiene should be performed after touching a patient’s surroundings, but this is difficult as residents are mobile, so the entire home may be their surroundings. It was therefore unsurprising that none of the learners could recall all 5 Moments and instead named other care tasks. Regarding gloves, only 52% correctly stated that hand hygiene should be practiced before and after wearing gloves, although most learners wear them to touch residents. This may be because healthcare workers sometimes practice hand hygiene only to protect themselves.^3^^,^^51^ Literature also explains that gloves may reduce hand hygiene adherence^52^ and are often worn incorrectly (eg, layered or sanitized)^53^ by care and nursing home staff. In a study of 20 nursing homes in Norway, compliance dropped by 30.8% when wearing gloves and was not performed correctly in 64.7% of instances.^51^ Similarly, gloves were appropriately used in just 16.8% of observations by personal care staff in Hong Kong, compared to 54.7% by professional staff (primarily nurses).^52^

### Implications

Care home managers should consider rolling out AR/VR hand hygiene training, supported by workforce training policies. Further experimental research should determine whether AR/VR training is more effective than previous training, any long-term impacts, and how many sessions are required for optimal learning. As the non-immersive VR training was unacceptable, usability improvements are needed, such as simplifying navigation. Other technical improvements include amending the VR scenarios to be care home related and completing the AR training on an iPad/tablet to minimize screen size issues.

### Limitations and strengths

There were only 3 repetitions within the training, so our ability to determine how many repetitions are required to support and sustain learning is limited. Another potential limitation is selection bias, as participants may have been more comfortable with new technologies or digitally literate. Lastly, we did not formally assess cost-effectiveness, which is an important factor to consider regarding the feasibility and future implementation of the training.

Methods, analyst, and data triangulation were a strength. The questionnaires, interviews, and observations explored many of the same phenomena. Two researchers individually coded the qualitative data which were collected from different care homes and learners. The representativeness of our study sample also strengthens the generalizability of our findings to other care homes in the United Kingdom.

## Conclusion

AR and immersive VR smartphone apps are feasible, usable, and acceptable technologies for delivering more engaging and potentially effective hand hygiene training in care homes. Future work that explores whether hand hygiene training delivered via AR/VR is more effective than previous training is warranted. There is also a need to improve the non-immersive VR and ensure roll-out of the training includes the AR training as the non-immersive alternative, to provide equitable training opportunities.

## Supplementary Material

ocad200_Supplementary_Data

## Data Availability

The data underlying this article cannot be shared due to ethical reasons.
