# Supplementary material for: Virtual reality and augmented reality smartphone applications for upskilling care home workers in hand hygiene: a realist multi-site feasibility, usability, acceptability, and efficacy study
Source: J Am Med Inform Assoc. 2023 Oct 16;31(1):45–60. doi: 10.1093/jamia/ocad200 (PMC10746305; doi:10.1093/jamia/ocad200)
Supplement: ocad200_Supplementary_Data [file ocad200_supplementary_data.docx]

**Supplementary file**

**INTERVIEW SCHEDULE**

1. Ask for consent to audio-record the interview
2. Refer to answers in the ‘Technology Acceptability’ questionnaire

- What did you think about the learning experience? How did it make you feel about your hand hygiene practice?
- Reflecting on your learning experience- do you think repeated practice is important for hand hygiene? Why/why not?
- Why (or why wasn’t) the task relevant to your work?
- To what extent did you think the tool was interactive? Was this important? Why/why not?
- To what extent was the technology able to remind you and give feedback on good hand hygiene practice? Was this important? Why/why not?
- Are there any context-related constraints we need to be aware of, when implementing the training?

**OBSERVATION SCHEDULE**

Throughout each session and immediately after, make comments on the form (as relevant)

| **Date:**  **Participant:**  **Start time:**  **End time:** | |
| --- | --- |
| **Prompts** | **Notes** |
| Challenges with setting up technology or running the study |  |
| Participant reactions toward technology/training |  |
| Challenges with using technology (participants) |  |
| Implementation barriers (e.g., cost, care home infrastructure, care home policies, tech ownership) |  |
| Implementation facilitators |  |
| Other observations |  |

**HAND HYGIENE IN HEALTH AND CARE SETTINGS KNOWLEDGE QUESTIONNAIRE

***adapted from the WHO Hand Hygiene in Health and Care Settings Brief Knowledge Questionnaire*

In this short survey we would like to know about your hand hygiene knowledge and confidence. Please answer the questions to the best of your ability. Your responses will remain anonymous. **Note:** Hand hygiene refers to hand washing (soap and water) OR hand rub (sanitizer use).

1. How long should hand hygiene be practiced for? *Select one answer*

- 3 seconds
- 30-40 seconds
- 1-2 minutes
- 10-20 seconds

1. When should health and care workers practice hand hygiene? List the key moments.
2. When should hand hygiene be practiced when wearing gloves?
3. Which of the following is the main route of cross-transmission of potentially harmful germs between patients in a health-care facility? (*tick one answer only*)

- Health-care workers’ hands when not clean
- Air circulating in the facility
- Patients’ exposure to colonised surfaces (i.e., beds, chairs, tables, floors)
- Sharing non-invasive objects (i.e., stethoscopes, pressure cuffs, etc.) between patients

1. When should you perform hand hygiene to prevent germs from spreading *to you*?
   *Select all that apply*

- After touching a resident/patient
- Immediately after a risk of body fluid exposure
- Immediately before a clean/aseptic procedure
- After exposure to the surroundings of a resident/patient

1. When should you perform hand hygiene to prevent spreading germs *to the resident*?
   *Select all that apply*

- Before touching a resident/patient
- Immediately after a risk of body fluid exposure
- After exposure to the surroundings of a resident/patient
- Immediately before a clean/aseptic procedure

1. Which of the following should be avoided, as they may increase germs on hands?
   *Select all that apply*

- Wearing jewellery
- Damaged skin
- Artificial fingernails
- Regular use of hand cream

**HAND HYGIENE CONFIDENCE**

*Circle the responses which best apply to you*

**1) I feel confident in my hand hygiene technique**

Strongly disagree. Disagree. Neither agree nor disagree. Agree. Strongly agree.

**2) My hand hygiene practice is up-to-date and aligns with best practice**

Strongly disagree. Disagree. Neither agree nor disagree. Agree. Strongly agree.

**3) I could improve my hand hygiene technique**

Strongly disagree. Disagree. Neither agree nor disagree. Agree. Strongly agree.

**4) I understand how and why infections spread**

Strongly disagree. Disagree. Neither agree nor disagree. Agree. Strongly agree.

**TECHNOLOGY ACCEPTABILITY AND TOLERABILITY***Circle the responses which best apply to you*

**1) The task in the learning tool was relevant to my work**

 Strongly disagree. Disagree. Neither agree nor disagree. Agree. Strongly agree.

**2) The learning tool is more interactive than previous training I have received on hand hygiene (e.g., videos, written descriptions, posters, pamphlets, verbal summaries)**

Strongly disagree. Disagree. Neither agree nor disagree. Agree. Strongly agree.

**3) The learning tool gave me feedback and reminded me about good hand hygiene practice**

Strongly disagree. Disagree. Neither agree nor disagree. Agree. Strongly agree.

**4) I would support further work of this technology in my training**

Strongly disagree. Disagree. Neither agree nor disagree. Agree. Strongly agree.

**5) Did you stop the training due to side effects (e.g., nausea, fatigue, dizziness, vertigo)?**

- No
- Yes

**6) During the training or immediately after, did you experience the following symptoms?***Select any that apply*

- None
- General discomfort
- Fatigue
- Headache
- Eye strain
- Difficulty concentrating
- Sweating
- Blurred vision
- Dizziness
- Stomach awareness (feeling of discomfort just short of nausea)
- Nausea
- Vertigo (loss of orientation with respect to being vertically upright)
- Other (please describe): ____________________________________________________

**Objective outcomes from the AR and VR apps**

**Compliance to the WHO 5 Moments for Hand Hygiene (VR training)**

The VR and non-immersive VR apps recorded compliance with the WHO 5 Moments for Hand Hygiene throughout the training. This showed an average score of 65% (±14.30) with an average of 3.79 missed opportunities of 11 (±1.59). Participants missed hand hygiene before an aseptic/clean procedure (97%, n=28), around wearing gloves (62%, n=18), after body fluid exposure/risk (55%, n=16), after touching a patient (55%, n=16) and before touching a patient (10%, n=3).

**Hand hygiene skill (AR training)**

The AR app recorded objective data on hand hygiene skill. The 19 participants reached proficiency at an average of 2.89 poses, including pose 1 (rub hands palm-to-palm), pose 2 (back of hands) and pose 3 (between fingers) (±0.81). All learners experienced difficulty with pose 6 (fingertips), 95% (n=18) had problems with pose 5 (thumbs), 84% (n=16) with pose 4 (backs of fingers) and 32% (n=6) with pose 3 (between fingers).
